# Rural Residents in China Are at Increased Risk of Exposure to Tick-Borne Pathogens *Anaplasma phagocytophilum* and *Ehrlichia chaffeensis*

**DOI:** 10.1155/2014/313867

**Published:** 2014-04-30

**Authors:** Lijuan Zhang, Hong Liu, Bianli Xu, Zhilun Zhang, Yuming Jin, Weiming Li, Qunying Lu, Liang Li, Litao Chang, Xiuchun Zhang, Desheng Fan, Minghua Cao, Manli Bao, Ying Zhang, Zengzhi Guan, Xueqin Cheng, Lina Tian, Shiwen Wang, Huilan Yu, Qiang Yu, Yong Wang, Yonggen Zhang, Xiaoyan Tang, Jieying Yin, Shijun Lao, Bin Wu, Juan Li, Weihong Li, Qiyi Xu, Yonglin Shi, Fang Huang

**Affiliations:** ^1^Department of Rickettsiology, National Institute for Communicable Disease Control and Prevention, China CDC, Changping, Beijing 102206, China; ^2^Centers for Disease Control and Prevention of Anhui Province, Hefei 650022, China; ^3^Centers for Disease Control and Prevention of Henan Province, Zhengzhou 450016, China; ^4^Tianjin Center for Disease Control and Prevention, Beijing 300011, China; ^5^Centers for Disease Control and Prevention of Hainan Province, Haikou 570203, China; ^6^Centers for Disease Control and Prevention of Jilin Province, Changchun 130062, China; ^7^Centers for Disease Control and Prevention of Zhejiang Province, Hangzhou 310051, China; ^8^Centers for Disease Control and Prevention of Jiangsu Province, Nanjing 210009, China; ^9^Centers for Disease Control and Prevention of Yunnan Province, Kunming 650022, China; ^10^Beijing Center for Disease Control and Prevention, Beijing 100013, China; ^11^YiLi Prefecture Center for Disease Control and Prevention, Yili 835000, China; ^12^Shihezi University, Shihezi 832000, China

## Abstract

As emerging tick born rickettsial diseases caused by *A. phagocytophilum* and *E. chaffeensis*, anaplasmosis and ehrlichiosis have become a serious threat to human and animal health throughout the world. In particular, in China, an unusual transmission of nosocomial cases of human granulocytic anaplasmosis occurred in Anhui Province in 2006 and more recent coinfection case of *A. phagocytophilum* and *E. chaffeensis* was documented in Shandong Province. Although the seroprevalence of human granulocytic anaplasmosis (former human granulocytic ehrlichiosis, HGE) has been documented in several studies, these data existed on local investigations, and also little data was reported on the seroprevalence of human monocytic ehrlichiosis (HME) in China. In this cross-sectional epidemiological study, indirect immunofluorescence antibody assay (IFA) proposed by WHO was used to detect *A. phagocytophilum* and *E. chaffeensis* IgG antibodies for 7,322 serum samples from agrarian residents from 9 provinces/cities and 819 urban residents from 2 provinces. Our data showed that farmers were at substantially increased risk of exposure. However, even among urban residents, risk was considerable. Seroprevalence of HGA and HME occurred in diverse regions of the country and tended to be the highest in young adults. Many species of ticks were confirmed carrying *A. phagocytophilum* organisms in China while several kinds of domestic animals including dog, goats, sheep, cattle, horse, wild rabbit, and some small wild rodents were proposed to be the reservoir hosts of *A. phagocytophilum*. The broad distribution of vector and hosts of the *A. phagocytophilum* and *E. chaffeensis*, especially the relationship between the generalized susceptibility of vectors and reservoirs and the severity of the disease's clinical manifestations and the genetic variation of Chinese HGA isolates in China, is urgently needed to be further investigated.

## 1. Introduction

Anaplasmosis and ehrlichiosis are emerging tick-borne rickettsial diseases (TBRDs) caused by the obligate intracellular bacteria* Anaplasma phagocytophilum *and* Ehrlichia chaffeensis*, respectively [1–3]. These two rickettsiae are both in the family Anaplasmataceae but are in different genera. Both bacteria are transmitted by hard ticks, and certain wild rodents and animals are reservoirs. In China,* A. phagocytophilum* bacteria had been isolated from* Apodemus agrarius, Tscherskia triton,* and sheep, respectively, and these animals might be reservoirs hosts of* A. phagocytophilum* [4]. Moreover, a recent national investigation assessing the epidemiological status of* A. phagocytophilum* among domestic animals in 10 provinces/cities in China showed that some domestic animals including dogs, goats, and cattle might be important reservoirs hosts of* A. phagocytophilum* [5].

Although the diagnosis of anaplasmosis and ehrlichiosis is difficult, the annual numbers of infections reported throughout the world have steadily increased [6, 7] since the first recognition of* E. chaffeensis *in 1986 [8] and of* A. phagocytophilum *in 1990 [9]. Seroepidemiological data from the United States suggest that infection rates of* A. phagocytophilum *in endemic areas are as high as 15–36% [10, 11]. In China, an investigation in Tianjin City, one of the largest municipalities and the largest trade port in the northern part of China, revealed that the average seroprevalence in farmers was 8.8% in 2006 [12]. In the United States, the incidence rate of* E. chaffeensis *increases from 0.8 to 3.0 cases/10^5^/year during 2000–2007, the hospitalization rate is 49.0%, and the case-fatality rate is 1.9% [7]. A recent investigation assessing the seroepidemiological status of* E. chaffeensis* among rural residents in Beijing indicated that the seroprevalence was 16.4% [13].

However, both anaplasmosis and ehrlichiosis are often underrecognized in China because epidemiological, ecological, clinical, and microbiological information about these two bacteria is very limited, and both diseases are often misdiagnosed due to their clinical manifestation's similarity to hemorrhagic fever with renal syndrome (HFRS) [14–16]. A typical example of misdiagnosis is the unusual cluster of nosocomial transmission of human granulocytic anaplasmosis (HGA) in a hospital in Anhui province in 2006. The index patient was originally misdiagnosed with HFRS, and five relatives of the patient and four medical workers were secondarily infected with HGA due to contact with blood or respiratory secretions, while the index patient experienced extensive hemorrhage and underwent endotracheal intubation [17].

Despite serological, molecular, and even etiological evidence demonstrating the nationwide distribution of* A. phagocytophilum* infections in humans, domestic animals, ticks, and rodents [4, 12–19], large-scale laboratory-based serological investigations among rural residents who may be at an increased risk of occupational and residential exposure are limited. Thus, it is crucial to obtain epidemiological data on geographical, occupational, and residential risk factors that could increase disease exposure. Herein, a cross-sectional epidemiological study of people residing in rural and urban areas was undertaken during March–May, 2009.

## 2. Materials and Methods

### 2.1. Ethics Statement

The study and the collection protocol were approved by the China CDC Institutional Review Board (no. 201103). Written consent was obtained before the blood sampling of participants. Parents provided written informed consent on behalf of all child participants. Preparations of positive rabbit sera used for quality control of antigen slide in the study were produced by rabbit immunization, and all experimental procedures were conducted to conform to the National Institutes of Health Guide for Care and Use of Laboratory Animals (J. Derrell Clerk, Ed., National Academy Press, Washington, DC, USA, 1996.) The Animal Ethics Committee of the Chinese Center for Diseases Control and Prevention approved a document on the experimental procedures (201104).

### 2.2. Study Area and Time

The nine provinces Zhejiang, Anhui, Jiangsu, Henan, Yunnan, Hainan, Xinjiang, Jilin, and Heilongjiang and the two independent municipalities Beijing and Tianjin were chosen based on the availability of information on recorded rickettsial infections for each province/city from March to May, 2009. The investigation time and order for each province/city were determined based on the breeding peak of ticks in the local areas. For each province or city, three or five rural counties were selected based on geographic location, for example, the eastern, southern, western, northern, and central areas of each province, to identify the investigation sites. In the same way, three to five villages were chosen based on their geographic locations in each county.

### 2.3. Study Population

Considering the age distribution and the accordance of labor style, the family as unite was investigated and sampled. Local permanent residents were selected from among the local government-registered families; for example, families were selected based on the last digit (odd or even) of the registration number of their registered permanent residence. For each selected family, every individual, including spouses and children, was included in the study. A standard questionnaire was used according to the “Guideline for the Control and Prevention of Human Granulocytic Anaplasmosis” issued by the Ministry of Public Health of the People's Republic of China in 2008 (the Ministry of Public Health of the People's Republic of China, 2008, No. 18). The demographic data collected included general information, such as sex, age, place of residence (plain areas or hilly regions), occupation (planting crops, planting fruit trees, or the unemployed, including retired people, students, and preschool children), the length of working time per day, the length of service time, and past medical history. The participants had to answer whether they could recognize ticks, whether they had been bitten by ticks, and how frequently they had been bitten by ticks within the last year. All participants were asked whether they had a fever on the day of the survey and whether they had had a fever (temperature of ≥38.0°C) during the preceding 12 months. If so, they were asked about clinical manifestations, such as myalgia or headache.

In addition to the rural residents mentioned above, a total of 819 samples from urban residents, including 566 sera from Daqing city, Heilongjiang province, and 253 sera from Yanbian city, Jilin province, were collected during 2007-2008 and included in the study. The demographic data were recorded in the same way as for the rural residents.

### 2.4. Sampling and Laboratory Detection

A 2 mL sample of nonanticoagulated blood was collected from each participant after written informed consent was obtained. Samples were temporarily stored in a cooler and then transferred to the local county CDC for serum separation. The blood samples were centrifuged at 1,500 rpm for 10 min, and the separated sera were stored at −20 or −40°C at the local CDC and then transferred to the Department of Rickettsiology, National Institute for Communicable Disease Control and Prevention, China CDC, by air within 48 or 72 hours and stored at −80°C until laboratory testing.

Immunofluorescence assays (IFA) were performed for IgG antibody detection according to the reference methods proposed [20]. To reduce laboratory errors, testing of all samples was performed within a limited time frame (from May to August 2009).* E. chaffeensis* (Arkansas strain) antigen was provided by Dr. Robert Massung at the United States CDC. The* A. phagocytophilum* strain Webster was kindly provided by J. S. Dumler at the Johns Hopkins University School of Medicine. These two antigens were spotted in different rows in the same slide to reduce laboratory errors.

HGA-positive human serum from the Focus Diagnostics kit (Cypress, CA) served as a positive control for testing human sera, and three or five different diluted concentrations of rabbit sera were used for quality control of antigen slide because of the limited HGA-positive human sera. Rabbit sera against* A. phagocytophilum* and* E. chaffeensis *were prepared by immunizing rabbits with* A. phagocytophilum* (Webster strain) and* E. chaffeensis *(Arkansas strain), respectively. These two positive rabbit sera were serially twofold diluted and then assayed in parallel with human HGA-positive control sera from a Focus Diagnostics kit using the antigen slice prepared by our laboratory and a slide from Focus, respectively. Based on the quality control methods recommended by Focus Diagnostics, three or five different concentrations of diluted rabbit sera were screened as positive controls. The rabbit sera were frequently standardized using the Focus Diagnostics kit, especially when new batches of antigens were prepared. Two negative controls were selected for each IFA: PBS-milk and mixed healthy human sera (from five workers at our institute who were not members of our laboratory).

The IFAs were performed as follows. The serum samples were diluted 1 : 80 in PBS containing 3% nonfat powdered milk, and 25 *μ*L of the diluted serum was placed in a slide well and incubated for 60 min in a moist chamber at 37°C. After washing in PBS to remove unbound antibody, the slides were labeled with FITC-conjugated rabbit anti-human immunoglobulin (IgG; Sigma Co., NY, New York State, United States) as a secondary antibody, which was diluted 1 : 400 with Evans blue, for another 60 min in a moist chamber at 37°C. The slides were then washed in PBS to remove unbound secondary antibody. The slides were air dried at 37°C and examined using a fluorescent microscope (Nikon, Tokyo, Japan). Samples were interpreted as reactive if there was strong green fluorescence corresponding to bacterial morulae within the cells on the slide. Samples reactive at the 1 : 80 screening dilution were considered to be positive [10, 12, 13]. If a serum sample had dual reactivity with* A. phagocytophilum* and* E. chaffeensis*, further dilution and titration were conducted, and a twofold or higher titer increase was read as positive.

### 2.5. Statistical Analysis

Statistical analysis was conducted using SAS software (version 9.1, SAS Institute, Inc., Cary, NC). Age was converted into a categorical variable (<10, 10–19, 20–29, 30–39, 40–49, 50–59, and >60 years of age). *χ* ^2^ and Fisher's tests were used to compare distributions of seropositivity or to examine associations between pairs of categorical measures. Logistic regression analyses were used to calculate odds ratios (ORs) for seropositivity among variables. The survey questions regarding the variables “living in plains areas,” “living in hilly region,” “crop planting,” “planting fruit trees,” “livestock breeding or contact with domestic animals,” “length of working hours per day,” and “length of service time” were created to be associated with presumed risk among permanent residents of rural areas andto develop the explanatory variables used in the logistic regression. All tests were two-sided, and significance was set at less than 0.05.

## 3. Results

### 3.1. Study Areas and Population

**Figure 1:**
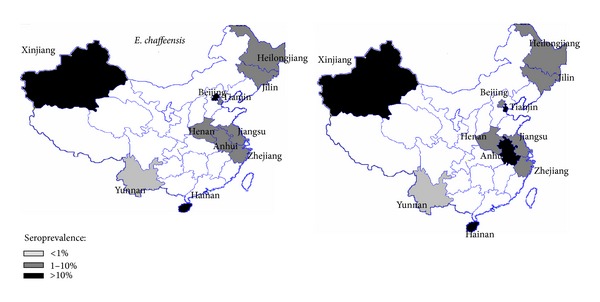
The seroprevalence distribution of* E. chaffeensis* and* A. phagocytophilum* from 11 provinces/cities in China from 2007 to 2009. The triangles on the map indicate the investigated counties in each province.

A total of 7,322 rural residents from 57 villages in 33 rural counties in Zhejiang, Anhui, Jiangsu, Henan, Yunnan, Hainan, and Xinjiang provinces and from the cities of Beijing and Tianjin were investigated during March–May, 2009 (Figure 1). The mean age of the participants was 44 years (range, 2–81 years). Males accounted for 3,493 of the participants, with a mean age of 43 years (range, 2–80 years), and females accounted for 3,829 of the participants, with a mean age of 45 years (range, 2–81 years). In the rural areas investigated, 72% of participants lived in plains areas, and 28% of people lived in hilly regions. In total, 57% of residents were engaged in planting crops; 23% of residents were engaged in planting fruit; 4% of residents were engaged in feeding domestic animals; and 16% of investigated individuals were preschool children, students, or retired people. Additionally, 95% of residents had contact with domestic animals or livestock. Although 94% of people could recognize ticks and 12% of people recalled that they had been bitten by ticks before, nobody could tell the species of the ticks. In total, 6% of residents recalled fever (≥38°C), headache, or myalgia during the past year. Only 15% (65) of people remembered the name of the antibiotics that they had used, for example, a tetracycline (34), penicillin (15), a cephalosporin (13), or a macrolide (3).

The characteristics of the 819 urban residents were as follows: 310 sera (from males aged 18–25 years) were collected from people who were ready to be drafted into the army in Daqing city in 2007, Heilongjiang province, 98% of whom were senior high school students, and 2% of whom were young people waiting for job assignments. Another 256 sera (from females aged 23–67 years) from Daqing were collected from healthy people who had participated in a medical examination in 2008. In addition, 253 sera (from 121 males aged 18–76 years and 132 females aged 20–83 years) were collected from healthy enterprise employees at a medical examination center in Yanbian city, Jilin province, in 2008. All of the people mentioned above lived in cities and rarely engaged in work in the wild or were exposed to ticks.

### 3.2. Seroprevalence of* E. chaffeensis*

Values for the seroprevalence of* A. phagocytophilum *and* E. chaffeensis* in the studied areas are shown in Table 1 and Figure 1. The overall seroprevalence of* E. chaffeensis* was 9.8% (95% confidence interval (CI) 9.1–10.5%) in rural residents and 2.4% (95% CI 1.4–3.4%) in urban residents. Chi-square test analysis indicated that the seropositivity rates of rural residents were significantly higher than the rates of urban residents (*P* = 0.0001, OR 4.4, 95% CI 2.8–6.8%). In an IFA, 37 (0.45%) samples exhibited a cross-reaction with* A. phagocytophilum *and* E. chaffeensis* at a 1 : 80 cut-off, 16 (43%, 16/37) of which were confirmed to be reactive with* E. chaffeensis* by further titration. Among the seven provinces and two cities, the seropositivity rates of* E. chaffeensis *of rural residents in Xinjiang (43.2%, 95% CI 28.6–57.8%) and Hainan (44.6%, 95% CI 41.3–47.9%) provinces and Beijing city (19.4%, 95% CI 9.9–28.9%) were significantly higher compared with rates in other areas. The difference in serological prevalence between males and females in each province or city was not statistically significant (Table 2). Although age distribution differed across age strata, the seroprevalence in the 20-to-29-year-old (15.3%, 95% CI 13.5–17.1%) and 30-to-39-year-old (13.0%, 95% CI 11.4–14.6%) groups was significantly higher than in the other age groups (*P* < 0.05) (Table 3). However, no statistically significant difference was found between the two groups mentioned above (*P* = 0.06). Of the seven provinces and two cities in rural areas, the seroprevalence (44.6%, 95% CI 41.3–47.9%) was the highest in Hainan, followed by Xinjiang (43.2%, 95% CI 28.6–57.8%) and Beijing (19.4%, 95% CI 9.9–28.9%) (Table 2).

Regarding the variables associated with the presumed risk based on the questionnaires, a statistical analysis indicated that fever in the last 24 months and service time >2 years were associated with the exposure risk of* E. chaffeensis*. However, no association between seroprevalence and other specific demographic variables was observed (Table 4).

### 3.3. Seroprevalence of* A. phagocytophilum*

The overall seroprevalence of* A. phagocytophilum *was 15.4% (95% CI 14.6–16.2%) in rural residents and 1.5% (95% CI 0.7–2.3%) in urban residents. The seroprevalence in rural residents was significantly higher than in urban residents (*P* < 0.0001, OR 12.7, 95% CI 7.2–22.5%). The seroprevalence varied between investigated sites, and the highest seroprevalence (41.8%, 95% CI 38.5–45.1%) of* A. phagocytophilum *was in Tianjin, followed by Hainan (39.2%, 95% CI 35.9–42.5%), Anhui (33.7%, 95% CI 39.5–40.2%), and Beijing (13.6%, 95% CI 3.5–23.7%) (Table 2). Analysis of sex indicated that the total seroprevalence of* A. phagocytophilum *in males (21.4%, 95% CI 20.0–22.8%) was significantly higher than in females (15.2%, 95% CI 14.1–16.3%) (*P* < 0.001, OR 1.5, 95% CI 1.3–1.7%). Similarly, the seroprevalence of* A. phagocytophilum* in the 20-to-29-year-old (20.2%, 95% CI 18.2–22.2%) and 30-to-39-year-old (25.3%, 95% CI 23.2–27.4%) groups was higher than in the other age groups (*P* < 0.05), although the seroprevalence varied between individual age groups.

Regarding associations between demographic characteristics and seropositivity, our data showed that the seroprevalence in residents who were engaged in planting crops was significantly higher than in people who were employed predominantly in fruit tree planting (*P* = 0.006, OR 0.8, 95% CI 0.7–0.9%). Similarly, the seroprevalence in residents who had contact with domestic and livestock animals was significantly higher than in residents without contact with animals (*P* < 0.0001, OR 4.0, 95% CI 2.6–6.3%). In addition, the seroprevalence in people who had worked for more than 2 years was higher than in people who had worked for less than 2 years (*P* < 0.0001, OR 1.8, 95% CI 1.5–2.1%). Additionally, fever in the last 24 months was associated with a high seroprevalence of* A. phagocytophilum *(*P* < 0.0001, OR 0.04, 95% CI 0.03-0.04%). However, no association was observed between tick bites and human infection, although tick exposure and bites were major risk factors for* A. phagocytophilum *and* E. chaffeensis* infections (*P* = 0.3, OR 1.1, 95% CI 0.9–1.3%).

### 3.4. Comparative Distribution of* A. phagocytophilum* and* E. chaffeensis*

Comparing the distribution of* A. phagocytophilum* and* E. chaffeensis*, the seroprevalence (33.7%, 95% CI 39.5–40.2%) of* A. phagocytophilum *in Anhui was strikingly higher than that of* E. chaffeensis *(5.5%, 95% CI 3.8–7.3%) (OR 0.12, 95% CI 0.08–0.2%). The same tendency was observed in Tianjin (OR 0.14, 95% CI 0.2–0.2%) (Table 5). In contrast, the seroprevalence of* E. chaffeensis *in Hainan (44.6% versus 39.2%, *P* = 0.03, OR 1.2, 95% CI 1.0–1.5%) and Xinjiang (43.2% versus 13.6%, *P* = 0.002, OR 4.8, 95% CI 1.7–13.7%) provinces and in Beijing city (19.4% versus 14.1%, *P* = 0.02, OR 1.5, 95% CI 1.1–2.0%) was significantly higher than that of* A. phagocytophilum *(Table 5).

## 4. Discussion

Regarding emerging zoonotic infectious diseases, increasing numbers of HGA cases have been confirmed in China since the unusual transmission of nosocomial cases of HGA occurred in Anhui province in 2006 [14, 16, 21]. Specifically, a case of coinfection with* A. phagocytophilum* and* E. chaffeensis* was reported in Shandong province [22]. A recent nationwide etiological investigation of HGA indicated that a total of 46 confirmed and 16 probable HGA cases were recorded from 2009 to 2010, and these cases were broadly distributed in Hebei, Shandong, and Henan provinces and in Beijing and Tianjin cities [21]. In this report, 41.2% of patients were diagnosed with multiple organ dysfunction syndrome (MODS), and the fatality rate was as high as 8.1%. Four human HGA isolates were obtained from patients, and one tick isolate was obtained from the* Haemaphysalis longicornis* parasite on the bodies of the domestic animals owned by these patients. Among these HGA isolates, two human isolates and one tick isolate from Shandong Peninsula, where all of the patients exhibited severe clinical manifestations, were identical to each other, based on an analysis of 16S rRNA and the* ankA *and* msp2*  genes but were different in sequence from sequences identified in other parts of the world. Moreover, the 16S rRNA gene of the five Chinese HGA isolates showed 99% identity with the strain China-C-Tt (GQ 412339) in* Tscherskia triton*, the strain China-C-Y (GQ412338) in domestic sheep, and the strain China-C-Aa (GQ 412337) in* Apodemus agrarius* from the northeastern areas of China [4]. Here, we had to address that some genetic groups of* A. phagocytophilum* identified in China were related to human infection, while others might be only associated with sylvatic or domestic animals but not able to infect humans. However, these nonpathogenic Anaplasma such as* A. platys*,* A. ovis,* and* A. bovis* might inflate the seropositivity in the study. Similarly, some isolates of* E. chaffeensis* and other related organisms such as* E. canis* that may not be pathogenic to humans might elicit anti-*Ehrlichia* antibodies. In addition, the genetic diversity of the key immunogenicity MSP2 proteins between Chinese HGA isolates and USA HGA isolates mentioned above might impact the seropositivity in the study.

In China, the free-range feeding of animals is a major part of livestock production, in contrast to livestock production in modern developed countries. Animals roam hills for feeding during daylight and return at sundown. In such a situation, animals can return with many ticks from wild fields. Moreover, most farm families own 2-3 dogs for guarding their animals and belongings, and these dogs also roam freely in and out of yards. Therefore, it is not surprise that contacting with domestic animals is regarded as a main exposure risk of* A. phagocytophilum*. A national investigation on domestic animals in 10 provinces/cities of China indicated that the PCR-positive rates for* A. phagocytophilum *16S rRNA were 26.7% for goats, 23.4% for cattle, and 10.9% for dogs [5].

Phylogenetic analyses of the 16S rRNA genes identified in these animals and ticks indicated that the dominant group, which consisted of 59.2% of the sequences from the domestic animals and 67% of the sequences from the ticks, was grouped with the sequences of the two human Chinese HGA isolates from Shandong province, mentioned above [21]. Moreover, the sequences (EF211110) identified in a patient with a nosocomial infection in Anhui in 2006 and the sequences (EU982709) from a patient in Yiyuan county, Shandong province, in 2007, were classified into the group [5].

The geographic distribution of* A. phagocytophilum* was mainly in Hainan, Anhui, Tianjin, and Beijing (Table 2). Hainan province is the second largest island in China, and its climate characteristics are advantageous to vector-borne rickettsial diseases. A retrospective field investigation on rickettsioses in Chengmai county, Hainan province, revealed that 5% of farmers' houses contained ticks, and a tick blood trail could be observed on the walls of the houses. Additionally, 15% of local children (40/270) had a typical eschar or rash on their bodies. A total of 11 isolates of rickettsiae were isolated from 23 febrile patients, and seven isolates of rickettsiae were isolated from wild* Rattus flavescent,* which were captured in local areas. The field investigation indicated that the seroprevalence was 6.3% (51/812) for* A. phagocytophilum*, 12.5% (101/812) for* E. chaffeensis,* and 37.5% (305/812) for spotted fever rickettsia in the local population [23]. PCR amplification of the 16S rRNA genes of rickettsiae in tick samples indicated that the positive rates were 23.3% (7/30 sample pools) for* R. sanguineus*, 6.9% (2/29 sample pools) for* H. doenitzi, *and 12.7% (9/71 sample pools) for* R. microplus *[23]. Anhui province is located in the middle of the eastern part of China and an unusual outbreak of anaplasmosis occurred in a hospital in this province in 2006. In a previous study, we focused on investigating Guangde county, where the index patient from the nosocomial transmission of HGA lived; Huaiyuan county; and Mingguang city. The results demonstrated that the average seropositivity rate of* A. phagocytophilum* among rural residents was 33.7% (201/596) [24]. Of the three sites investigated, Guangde county had the highest seroprevalence (76.5%, 153/200) and Huaiyuan county had the lowest (10.4%, 26/249). Tianjin CDC conducted a continuous seroepidemiological investigation of* A. phagocytophilum* among people at high risk of exposure (animal breeders, hand-milking workers, animal birth assistants, and cleaners) from 2006 to 2009. The results indicated that the total seroprevalence of* A. phagocytophilum* was 8.8% in 2006 [12], 44.4% (169/381) in 2007, 42.9% (110/256) in 2008, and 59.2% (147/249) in 2009 [25]. We proposed that this dramatic change might be associated with the different occupational structure of the participants each year (from ordinary rural residents in 2006 to people at high risk of exposure from 2007 to 2009). Another reason for such changes might be related to the different natural geographic characteristics of the sites investigated each year (from high-altitude areas in 2006 to low-altitude areas from 2007 to 2009). A recent etiological investigation of HGA in Tianjin revealed four laboratory-probable HGA cases among 24 undiagnosed febrile patients [21].

Because human monocytic ehrlichiosis (HME) is an emerging tick-borne infectious disease, seroepidemiological information about HME is limited in China. This study serologically demonstrated a higher prevalence rate of* E. chaffeensis* among farm residents in Hainan (44.6%, 95% CI 41.3–47.9%), Xinjiang (43.2%, 95% CI 28.6–57.8%), and Beijing (19.4%, 95% CI 9.9–28.9%) (Table 2). Additionally, Hainan and Xinjiang provinces shared a higher coprevalence of* E. chaffeensis *and* A. phagocytophilum.* Xinjiang province, located in central Asia and neighboring Mongolia, Russia, and Kazakhstan, is famous in China for numerous tick species. Many studies have identified* E. chaffeensis* in ticks collected from Xinjiang province [26] and other parts of China [27] and from countries neighboring China [28].

The sex distribution varied between the areas investigated, but male farm residents are at a higher risk of infection with* A. phagocytophilum *than females when considering the total population studied, although no differences were observed between males and females for* E. chaffeensis*. An analysis of the age distribution indicated that the highest seroprevalence of* E. chaffeensis *and* A. phagocytophilum *was identified in the 20-to-29-year-old and 30-to-39-year-old groups (Table 3). We presumed that this phenomenon was due to more outside activities among these young people.

Our current findings and previous data provide strong evidence that* A. phagocytophilum *and* E. chaffeensis* exist in China [4, 5, 12–19, 21–25, 29, 30] and in other Asian countries [28, 31, 32]. Not only rural residents but also urban residents were at a substantially increased risk of exposure to these pathogens. Although the seroprevalence of urban residents were lower than that of rural residents, we had to address that the results in the study might be affected by the geographic characterizes [33, 34]. The urban samples were collected from Daqing city of Heilongjiang and Yanbian city of Jilin provinces, which located in the northeast of China and shared cooler climate, especially Daqing areas characterized with sterile saline and alkali soil with rare vegetative cover was not conductive to the breeding of ticks.

More and more researches indicated that different climate and other environmental conditions determined the distribution of tick-borne pathogens [33, 34]. China is one of the largest countries in the world and had complex ecological system and each province had different geographic and climatic characteristics. It is reported that Xinjiang province alone had more than 50 species of ticks [35].

For analysis of proposal transmission vector of* A. phagocytophilum* in China, an epidemiological field investigation found that there were many ticks on the bodies of animals, and at least six species of ticks were identified. PCR amplification of* A. phagocytophilum *16S rRNA showed that the positive rates were 58.3% for* Dermacentor silvarum*, 43.9% for* Haemaphysalis longicornis*, 12.5% for* Ixodes persulcatus*, 7.5% for* Rhipicephalus microplus,* and 5.2% for* Rhipicephalus sanguineus* [5]. More molecular investigations indicated that* Ixodes persulcatus, Dermacentor silvarum, Haemaphysalis concinna, Haemaphysalis longicornis, Rhipicephalus microplus, Rhipicephalus sanguineus*, and* Dermacentor nuttalli* might transmission of* A. phagocytophilum* in China [5, 23, 27, 29, 36]. Notably, such more species of ticks carrying* A. phagocytophilum* organisms in China were different from those found in USA. Whether this phenomenon was associated with some genetic variations of Chinese HGA isolates mentioned above remained to be further explored.

Additionally, some recent researches showed that several kinds of domestic animals including dog, goats, sheep, cattle, horse, wild rabbit, and some small wild rodents including* Apodemus agrarius*,* Tamias sibiricus, Apodemus peninsulae, Apodemus sylvaticus, Clethrionomys rufocanus, Niviventer confucianus, Niviventer coxingi, Niviventer anderson, Niviventer eha, Rattus nitidus, Al XII temus latronum, Apodemus chevrien, Apodemus draco, Eothenomys eleusis, Eothenomys custos, Eothenomys cachinus, Tamiops swinhoei, *and* Naaillus gracilis *were proposed to be the reservoir hosts of* A. phagocytophilum* [18, 19, 23, 24, 27–29, 37, 38].

Although the epidemiology data of* E. chaffeensis* is very limited in China, a case of coinfection with* A. phagocytophilum* and* E. chaffeensis* was documented in Shandong province [22]. Furthermore, 3.84% of coinfection rates of* A. phagocytophilum* and* E. chaffeensis* were found in* Gerbillus* sp. collected from Xinjiang province, which is the biggest desert in China where the* Gerbillus* spp. are the dominant rat [39]. Previously studies and recent investigations revealed that several species of hard ticks including* Ixodes persulcatus, Dermacentor silvarum, *and* Dermacentor nuttalli *[40] and* Rhipicephalus microplus* [41] might be associated with the transmission of* E. chaffeensis* in China.

As emerging tick born infection diseases, the distribution of vector and hosts of the* A. phagocytophilum *and* E. chaffeensis* and their role in the transmission of these pathogens are limited. Such information is urgently needed to be further investigated to better understand the pathogenesis of these pathogens, especially the relationship between the generalized susceptibility of vectors and reservoirs, the severity of the disease's clinical manifestations, and the genetic variation of Chinese HGA isolates in China.

In summary, the tick born rickettsial diseases caused by* A. phagocytophilum* and* E. chaffeensis* have become a serious threat to human and animal health. Several measures should be taken to minimize the likelihood of becoming infected with these two zoonotic rickettsiae from direct contact with farm animals, especially among individuals who work with livestock. The health management department should emphasize the differentiation of these zoonotic infectious diseases from other febrile diseases, especially for the prevention and control of nosocomial human-to-human transmission, during treatment.

## Figures and Tables

**Table 1:** The overall seroprevalence of *E. chaffeensis* and *A. phagocytophilum* in rural and urban residents, China, 2007–2009.

Pathogens	Rural		Urban		Odds ratio (95% CI)	*P* value
	Number positives/total number	Seropositivity rate % (95% CI)	Number positives/total number	Seropositivity rate % (95% CI)		
*E. chaffeensis *	719/7322	9.8 (9.1–10.5)	20/819	2.4 (1.4–3.4)	4.4 (2.8–6.8)	<0.0001
*A. phagocytophilum *	1331/7321	15.4 (14.6–16.2)	12/819	1.5 (0.7–2.3)	12.7 (7.2–22.5)	<0.0001

CI: confidence interval.

**Table 2:** The seroprevalence of *E. chaffeensis* and *A. phagocytophilum *by location and sex.

Area	Province/ sampling year	*E. chaffeensis *						*A. phagocytophilum *					
		M		F		Odds ratio (95% CI)	*P* value	M		F		Odds ratio (95% CI)	*P* value
		Number positives/total number	Seropositivity rate % (95% CI)	Number positives/total number	Seropositivity rate % (95% CI)			Number positives/total number	Seropositivity rate % (95% CI)	Number positives/total number	Seropositivity rate % (95% CI)		
Rural	Anhui/2009	13/244	5.3 (2.5–8.1)	20/352	5.7 (3.2–8.1)	0.9 (0.4–1.9)	0.9	101/244	41.4 (35.2–47.6)	100/352	28.4 (23.7–33.1)	1.8 (1.3–2.5)	0.001
	Hainan/2008	186/426	43.7 (35.8–51. 9)	193/424	45.5 (39.8–51.2)	0.9 (0.7–1.2)	0.6	172/427	40.3 (35.6–45.0)	162/425	38.1 (33.5–42.7)	1.1 (0.8–1.4)	0.5
	Henan/2009	21/348	6.0 (3.5–8.5)	26/389	6.7 (4.2–9.2)	0.9 (0.5–1.6)	0.7	30/348	8.6 (5.7–11.5)	36/389	9.3 (6.4–12.2)	0.9 (0.5–1.5)	0.8
	Jiangsu/2009	8/1161	0.7 (0.2–1.2)	33/1586	2.1 (1.4.–2.8)	3.6 (1.0–13.3)	0.08	143/1160	12.3 (10.4–14.2)	131/1586	8.3 (6.6–9.7)	1.5 (1.2–2.0)	0.0004
	Zhejiang/2009	3/279	1.1 (0.5–1.7)	4/300	1.3 (0.6–2.6)	0.8 (0.2–3.6)	1.0	1/279	0.4 (0.3–1.1)	2/300	0.7 (0.2–1.6)	0.5 (0.05–5.9)	1.0
	Yunnan/2009	1/151	0.7 (0.6–2.6)	0/175	0	0.5 (0.1–3.9)	0.5	0/151	0	1/176	0.6 (0.5–1.7)	1.0 (0.9–1.1)	1.0
	Xinjiang/2009	12/30	40.0 (22.5–57.5)	7/14	50.0 (23.8–76.2)	0.7 (0.2–2.4)	0.5	4/30	13.3 (7.9–20.1)	2/14	4.3 (10.5–16)	1.0 (0.1–5.7)	1.0
	Tianjin/2007, 2008/2009	45/581	7.7 (5.5–9.9)	38/301	12.6 (8.9–16.3)	0.6 (0.4–0.9)	0.02	253/578	43.8 (39.8–47.8)	114/301	37.9 (32.4–43.4)	1.3 (1.0–1.7)	0.09
	Beijing/2009	50/273	18.3 (13.7–22.9)	59/288	20.5 (15.8–25.2)	0.9 (0.6–1.3)	0.5	43/273	15.8 (11.5–20.1)	36/288	12.5 (8.6–16.3)	1.3 (0.8–2.1)	0.3
	Total	**339/3493**	**9.7 (8.7–10.7)**	**380/3829**	**9.9 (9.0–10.8)**	**1.0 (0.8–1.1)**	**0.8**	**747/3490**	**21.4 (20.0–22.8)**	**584/3831**	**15.2 (14.1–16.3)**	**1.5 (1.3–1.7)**	**<0.0001**

Urban	Jilin/2008	5/106	4.7 (0.7–8.7)	11/147	7.5 (3. 2–11.8)	0.6 (0.2–1.8)	0.4	2/106	1.9 (0.7–4.5)	2/147	1.4 (0.5–3.3)	1.3 (0.1–10.0)	0.7
	Heilongjiang/2008	2/310	0.7 (0.2–1.6)	2/256	0.8 (0.3–1.9)	0.8 (0.1–5.9)	1.0	4/310	1.3 (0.1–2.6)	4/256	1.6 (0.1–3.1)	0.8 (0.2–3.3)	0.8
	Total	**7/416**	**1.7 (0.5–2.9)**	**13/403**	**3.2 (1.5–4.9)**	**0.5 (0.2–1.3)**	**0.2**	**6/416**	**1.4 (0.3–2.5)**	**6/403**	**1.5 (0.3–2.7)**	**0.9 (0.3–3.0)**	**1.0**

**Table 3:** The seroprevalence of *E. chaffeensis* and *A. phagocytophilum* by age.

Age	*E. chaffeensis *		*A. phagocytophilum *		OR (95% CI)	*P* value
	Number positives/total number	Seropositivity rate % (95% CI)	Number positives/total number	Seropositivity rate % (95% CI)		
Rural						
2–19	105/937	11.2 (9.2–13.2)	139/936	14.8 (12.5–17.1)	0.72 (0.6–1.0)	0.02
20–29	241/1574	15.3 (13.5–17.1)	318/1574	20.2 (18.2–22.2)	0.71 (0.6–0.9)	0.0003
30–39	217/1670	13.0 (11.4–14.6)	423/1670	25.3 (23.2–27.4)	0.44 (0.4–0.5)	<0.0001
40–49	147/1770	8.3 (7.0–9.6)	246/1770	13.9 (12.3–15.5)	0.56 (0.5–0.7)	<0.0001
50–59	70/1137	6.2 (4.8–7.6)	124/1137	10.9 (9.1–12.7)	0.5 (0.4–0.7)	<0.0001
>60	11/234	4.6 (1.9–7.3)	16/234	6.8 (3.7–10.0)	0.67 (0.3–1.5)	0.3
Urban						
18–29	5/370	1.3 (0.2–2.5)	4/370	1.1 (0–2.2)	1.3 (0.3–4.7)	1.0
30–39	3/138	2.1 (0.3–4.5)	3/138	2.1 (0.3–4.5)	1.0 (0.2–5.0)	1.0
40–49	7/136	5.1 (1.4–8.8)	3/136	2.2 (0.3–4.7)	2.4 (0.6–9.5)	0.2
50–59	3/98	3.0 (0.3–6.3)	1/98	1.0 (0.9–3.0)	3.0 (0.3–29.9)	0.6
>60	2/77	2.5 (1.0–6.0)	1/77	1.3 (1.2–3.8)	2.0 (0.2–22.8)	1.0

**Table 4:** Analysis of the presumed risk of *E. chaffeensis* and *A. phagocytophilum *among rural residents.

Variables	*E. chaffeensis *						*A. phagocytophilum *					
	Number (%) of residents						Number (%) of residents					
	Total cohort *N* = 7322	IFA positive N = 719	IFA negative N = 6603	OR	95% CI	P value	Total cohort N = 7321	IFA positive N = 1331	IFA negative N = 5990	OR	95% CI	P value
Living in plains areas	5271 (72.0)	506 (70.4)	4765 (72.2)	0.92	0.8–1.1	* *0.3	5271 (72.0)	945 (71.0)	4326 (72.2)	0.96	0.8–1.1	* *0.6
Living in hilly areas	2051 (28.0)	213 (29.6)	1545 (27.8)				2050 (28.0)	386 (29.0)	1664 (27.8)			
Planting crops	4174 (57.0)	396 (55.1)	3778 (57.2)	0.95^a^	0.8–1.1^a^	0.6^a^	4173 (57.0)	720 (54.1)	3452 (57.6)	0.8^a^	0.7–0.9^a^	0.006^a^
Planting fruit trees	1684 (23.0)	168 (23.4)	1516 (23.0)	0.90^b^	0.7–1.1^b^	0.2^b^	1683 (23.0)	342 (25.7)	1340 (22.4)	0.9^b^	0.8–1.1^b^	0.4^b^
Nonplanting	1464 (20.0)	155 (21.6)	1309 (19.8)	0.94^c^	0.7–1.2^c^	0.6^c^	1464 (20.0)	266 (20.0)	1198 (20.0)	1.1^c^	1.0–1.4^c^	0.1^c^
Contact with farm animals	6956 (95.0)	682 (94.8)	6274 (95.0)	0.97	0.71–1.4	0.9	6955 (95.0)	1311 (98.5)	5644 (94.2)	4.0	2.6–6.3	<0.0001
Tick bite in last 24 months	879 (12.0)	90 (12.5)	789 (11.9)	1.1	0.8–1.3	0.7	879 (12.0)	172 (12.9)	707 (11.8)	1.1	0.9–1.3	0.3
Fever in last 24 months	439 (6.0)	46 (6.4)	393 (6.0)	0.006	0.004–0.008	<0.0001	439 (6.0)	85 (6.4)	354 (5.9)	0.04	0.03–0.04	<0.0001
Working time >3 hours per day	5415 (74.0)	535 (74.4)	4882 (74.0)	1.0	0.9–1.2	0.8	5414 (74.0)	985 (74.0)	4429 (73.9)	1.0	0.9–1.2	1.0
Service time >2 years	5418 (74.0)	560 (77.9)	4858 (73.6)	1.3	1.1–1.5	0.01	5417 (74.0)	1095 (82.3)	4321 (72.1)	1.8	1.5–2.1	<0.0001

^a^Planting crops versus planting fruit trees.

^b^Planting crops versus nonplanting.

^c^Planting fruit trees versus nonplanting.

**Table 5:** Comparison of distribution of *E. chaffeensis* and *A. phagocytophilum *among areas in the study.

Area	Sites	*E. chaffeensis *		*A. phagocytophilum *		OR (95% CI)	*P* value
		Number positives/total number.	Seropositivity rate% (95% CI)	Number positives/total number	Seropositivity rate % (95% CI)		
Rural	Anhui	33/596	5.5 (3.8–7.3)	201/596	33.7 (39.5–40.2)	0.12 (0.08–0.2)	<0.0001
	Hainan	379/850	44.6 (41.3–47.9)	337/852	39.2 (35.9–42.5)	1.2 (1.0–1.5)	0.02
	Henan	47/737	6.4 (4.6–8.2)	66/737	9.0 (6.9–11.1)	0.69 (0.5–1.0)	0.06
	Jiangsu	41/2474	1.5 (1.0–2.0)	274/2473	10.0 (8.9–11.1)	0.14 (0.1–0.19)	<0.0001
	Zhejiang	7/579	1.2 (0.3–2.1)	3/579	0.5 (0.1–1.1)	2.3 (0.6–9.1)	0.2
	Yunnan	1/326	0.3 (0.29–0.9)	1/327	0.3 (0.29–0.9)	1.0 (0.1–16.1)	1.0
	Xinjiang	19/44	43.2 (28.6–57.8)	6/44	13.6 (3.5–23.7)	4.8 (1.7–13.7)	0.002
	Tianjin	83/882	9.4 (7.5–11.3)	367/879	41.8 (38.5–45.1)	0.14 (0.1–0.2)	<0.0001
	Beijing	109/561	19.4 (9.9–28.9)	79/561	14.1 (11.2–17.0)	1.5 (1.1–2.0)	0.02
	Total	**719/7322**	**9.8 (9.1–10.5)**	**747/3490**	**15.4 (14.6–16.2)**	**0.4 (0.4–0.5)**	**<0.0001**

Urban	Jilin	16/235	6.3 (3.3–9.3)	4/253	1.6 (0.1–3.1)	4.2 (1.4–12.8)	0.006
	Heilongjiang	4/566	0.7 (0–1.4)	8/566	1.4 (0.4–2.4)	0.5 (0.1–1.7)	0.3
	Total	**20/819**	**2.4 (1.4–3.4)**	**12/819**	**1.5 (0.7–2.3)**	**1.7 (0.8–3.5)**	**0.2**
